# Fruit consumption among adults in Germany

**DOI:** 10.17886/RKI-GBE-2017-041

**Published:** 2017-06-14

**Authors:** Gert B. M. Mensink, Anja Schienkiewitz, Cornelia Lange

**Affiliations:** Robert Koch Institute, Department for Epidemiology and Health Monitoring, Berlin, Germany

**Keywords:** FRUIT, ADULTS, INTERVIEW SURVEY, HEALTH MONITORING, GERMANY

## Abstract

Eating fruit is part of a healthy diet and can help prevent various chronic diseases. According to GEDA 2014/2015-EHIS data, 54.2% of women and 38.1% of men eat fruit daily. 38.0% of women and 25.5% of men aged 18 to 29 years eat fruit daily; and in the age group of 65 and older this figure rises to 72.6% for women and 61.1% for men. In the age groups under 65, women with higher levels of education are more likely to eat fruit every day, for men this correlation applies only to those aged between 45 and 64. In Brandenburg, Mecklenburg-West Pomerania, Saxony, Saxony-Anhalt and Thuringia, the proportion of women and men who eat fruit daily is higher than the German average, and in Bavaria and Saarland the proportion of men who eat fruit daily is lower than the German average.

## Introduction

Fruit includes the edible fruits and seeds of mostly perennial plants. Like vegetables, there are many different types of fruit and due to global trade the market in Germany is continuously expanding. Fruit can be subdivided into groups such as pomaceous fruit, berries or citrus fruit. Whereas vegetables are usually eaten at meals, fruit is often a dessert or eaten as a snack between meals. Fruit is an important source of vitamins, minerals, trace elements, phytochemicals and fibre, yet has only little fat. Due to the variety of biologically active substances, eating fruit is associated with a number of health benefits. Beyond a high nutrient density, most fruit have high water contents and are therefore relatively low in calories [1]. Moreover, people who eat high quantities of fruit usually eat smaller amounts of physiologically less beneficial foods. A low energy content combined with high satiety means that eating lots of fruit and vegetables can contribute to maintain weight and to prevent obesity [1, 2].

Convincing evidence shows that eating high amounts of fruit and vegetables can help prevent, or positively influence the course, of coronary heart disease, high blood pressure and stroke [1, 3-5]. Probably, eating lots of fruit and vegetables also has a prophylactic effect on various cancers; the observed correlation with the overall cancer risk, however, is low [1, 6-9]. Consuming large amounts of fruit and vegetables is, according to a recent meta-analysis, associated with a lower overall mortality risk, in particular due to a lower cardiovascular mortality risk [8].

For some time now, this has been reflected in the implementation of various health-policy measures aimed at encouraging people to eat more fruit and vegetables.

**GEDA 2014/2015-EHIS**

**Data holder:** Robert Koch Institute

**Aims:** To provide reliable information about the population’s health status, health-related behaviour and health care in Germany, with the possibility of a European comparison

**Method:** Questionnaires completed on paper or online

**Population:** People aged 18 years and above with permanent residency in Germany

**Sampling:** Registry office sample; randomly selected individuals from 301 communities in Germany were invited to participate

**Participants:** 24,016 people (13,144 women; 10,872 men)

**Response rate:** 26.9%

**Study period:** November 2014 - July 2015

**Data protection:** This study was undertaken in strict accordance with the data protection regulations set out in the German Federal Data Protection Act and was approved by the German Federal Commissioner for Data Protection and Freedom of Information. Participation in the study was voluntary. The participants were fully informed about the study’s aims and content, and about data protection. All participants provided written informed consent.

More information in German is available at www.geda-studie.de

The ‘5 a day’ campaign, which recommends that people eat five portions of fruit and vegetables every day, is prob ably one of the most well-known. A portion of fruit or vegetables may occasionally be replaced by a smoothie or a glass of fruit or vegetable juice; however, the fruit or vegetable content of these drinks should be no less than 100%. A portion is defined as a handful of fruit or vege tables [10, 11].

## Indicator

Sufficient consumption of fruit and vegetables is a key element in a balanced and healthy diet. A population representative assessment of fruit consumption as an indicator of a healthy diet is therefore highly relevant for health policy. GEDA 2014/2015-EHIS assessed the frequency of people’s fruit consumption by asking: ‘How often do you eat fruit, including freshly pressed juices?’, with the possible answers ‘once or more a day’, ‘4 to 6 times a week’, ‘1 to 3 times a week’, ‘less than once a week’ and ‘never’. For the purpose of the analysis presented here, these answers were summarised into three categories (once or more a day, at least once a week and less than once a week). The results were stratified according to gender, age group, education and federal state. A statistically significant difference between groups is assumed when confidence intervals do not overlap.

The analyses are based on the data received from 23,947 participants aged 18 and above (13,104 women and 10,843 men) with valid information on fruit consumption. Calculations were carried out using a weighting factor that corrects for deviations within the sample from the German population (as of 31 December 2014) with regard to gender, age, district type and education. The district type accounts for the degree of urbanisation and reflects the regional distribution in Germany. The International Standard Classification for Education (ISCED) was used to improve the comparability of the responses provided on educational levels [12]. A detailed description of the methodology applied in the GEDA 2014/2015-EHIS study can be found in the article German Health Update – New data for Germany and Europe published in issue 1/2017 of the Journal of Health Monitoring.

## Results and discussion

The German Nutrition Society (DGE) recommends eating fruit and vegetables every day [10]. In Germany, many adults do not fulfil this recommendation. According to GEDA 2014/2015-EHIS, 54.2% of women and 38.1% of men eat fruit daily. Thus considerably more women than men eat fruit daily (Table 1 and Table 2). In the GEDA 2012 survey, 69.5% of women and 48.0% of men reported eating fruit daily [13]. Figures for vegetable consumption have also seen a similarly strong decline and this could be partly related to changes in the survey methodology (2014/2015: self-administered questionnaires; 2012: telephone interviews), as well as to different phrasing of questions and possible answers. GEDA 2012 respondents were asked on the telephone ‘How often do you eat fruit?’ and given the response options ‘every day’, ‘at least once a week’, ‘less than once a week’ and ‘never’. In GEDA 2014/2015-EHIS the question was formulated in writing as shown in the Indicator section. In GEDA 2012, people might have been inclined to answer ‘every day’ even if they only actually consumed fruit five or six times per week. The answer categories in GEDA 2012 may have partly led to the higher reported fruit consumption as compared to GEDA 2014/2015-EHIS.

**Figure 1:**
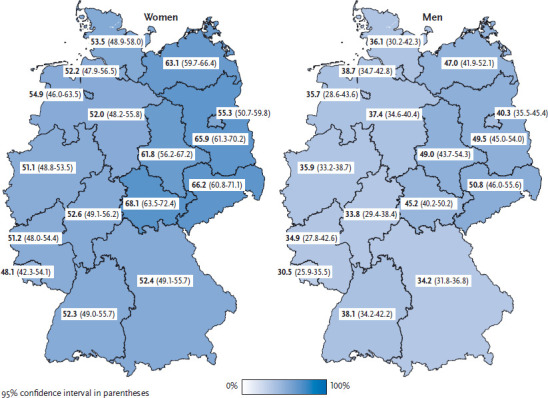
Daily fruit consumption according to gender and German federal state (n=13,104 women; n=10,843 men) Source: GEDA 2014/2015-EHIS

Daily consumption of fruit among women and men increases with age: Whereas only 38.0% of women and 25.5% of men aged 18 to 29 eat fruit daily, in the age group of 65 and older, the figures are 72.6% for women and 61.1% for men. Previous surveys also registered an increase in fruit consumption with age [13, 14]. Daily consumption of fruit is particularly widespread among the 65-years and older (Table 1 and Table 2). This could be due to the fact that people in this age group are more concerned both with their health and following a healthy diet. Moreover, they are less likely to be employed and, therefore, have more time to choose, buy and prepare their own food.They also cook more often every day or nearly every day than younger people [15]. In the age groups up to 65, women with higher levels of education eat significantly more often fruit every day. For men, a similar correlation between education and fruit consumption is seen only in the 45-to-65 age group. In Bavaria and Saarland, the proportion of men who consume fruit daily is significantly lower than the German average. In these states, no significant differences from the German average have been observed for women. In Brandenburg, Mecklenburg-West Pomerania, Saxony, Saxony-Anhalt and Thuringia, the proportion of women and men who consume fruit daily is significantly higher than the German average (Figure 1).

Compared with vegetable consumption, the percentage rates for daily fruit consumption are significantly higher. According to the German National Nutrition Survey II, in 2006, 54% of women and 65% of men did not consume the daily recommended amount of 250 g of fruit (not including juices) [16]. Results from the German Health Interview and Examination Survey for Adults (DEGS1) show that the mean fruit consumption is 1.8 portions for women and 1.2 portions for men per day, with 26.2% of women and 13.9% of men consuming fruit several times per day (excluding juices) [17]. Increasing the consumption of fruit remains desirable, in particular among men, young adults and people with low levels of education.

## Key statements

- 54% of women and 38% of men eat fruit daily.
- Daily fruit intake is higher with age.
- In the age groups up to 65, women with higher levels of education consume significantly more often fruit every day.

## Figures and Tables

**Table 1:** Fruit consumption among women according to age and educational status (n=13,104) Source: GEDA 2014/2015-EHIS

Women	Once or more a day		At least once a week		Less than once a week	
	%	(95% CI)	%	(95% CI)	%	(95% CI)
**Women total**	**54.2**	**(53.0-55.3)**	**38.5**	**(37.4-39.6)**	**7.3**	**(6.7-8.0)**
**18-29 Years**	38.0	(35.5-40.7)	50.8	(48.0-53.5)	11.2	(9.5-13.2)
Low education	33.7	(27.4-40.7)	50.7	(44.3-57.1)	15.6	(11.6-20.5)
Medium education	37.5	(34.3-40.8)	51.8	(48.3-55.3)	10.7	(8.6-13.3)
High education	47.1	(42.1-52.2)	46.2	(41.1-51.4)	6.7	(4.3-10.2)
**30-44 Years**	44.3	(42.0-46.6)	45.6	(43.3-47.9)	10.1	(8.7-11.7)
Low education	38.6	(32.0-45.6)	45.5	(38.2-52.9)	15.9	(11.3-22.0)
Medium education	40.6	(37.7-43.5)	48.4	(45.5-51.3)	11.1	(9.2-13.2)
High education	56.7	(53.6-59.8)	38.5	(35.6-41.6)	4.7	(3.4-6.6)
**45-64 Years**	53.7	(51.9-55.5)	39.5	(37.8-41.2)	6.8	(6.0-7.8)
Low education	52.8	(48.5-57.2)	38.0	(33.9-42.3)	9.2	(7.1-11.7)
Medium education	51.0	(48.7-53.3)	41.9	(39.7-44.2)	7.1	(5.9-8.5)
High education	63.5	(60.9-66.1)	32.6	(30.0-35.3)	3.9	(3.0-5.0)
**≥ 65 Years**	72.6	(70.5-74.7)	24.1	(22.3-26.1)	3.3	(2.5-4.3)
Low education	70.1	(66.4-73.4)	25.8	(22.6-29.2)	4.2	(2.8-6.2)
Medium education	73.7	(70.9-76.3)	23.5	(21.1-26.2)	2.8	(1.9-4.1)
High education	77.9	(73.1-82.0)	20.2	(16.1-25.0)	2.0	(1.0-4.0)
**Total (women and men)**	**46.3**	**(45.4-47.3)**	**43.1**	**(42.1-44.0)**	**10.6**	**(10.0-11.3)**

CI=confidence interval

**Table 2:** Fruit consumption among men according to age and educational status (n=10,843) Source: GEDA 2014/2015-EHIS

Men	Once or more a day		At least once a week		Less than once a week	
	%	(95% CI)	%	(95% CI)	%	(95% CI)
**Men total**	**38.1**	**(36.9-39.3)**	**47.8**	**(46.5-49.1)**	**14.1**	**(13.2-15.1)**
**18-29 Years**	25.5	(22.7-28.4)	55.7	(52.7-58.6)	18.9	(16.4-21.6)
Low education	24.7	(19.1-31.4)	53.1	(45.8-60.4)	22.1	(16.8-28.6)
Medium education	25.5	(22.1-29.2)	55.1	(51.4-58.7)	19.4	(16.4-22.8)
High education	25.8	(20.7-31.7)	63.6	(57.3-69.4)	10.6	(7.4-15.0)
**30-44 Years**	28.4	(26.2-30.8)	52.8	(50.0-55.6)	18.8	(16.5-21.2)
Low education	28.8	(21.6-37.2)	45.2	(36.6-54.1)	26.0	(19.3-34.1)
Medium education	26.5	(23.6-29.6)	53.1	(49.3-56.9)	20.3	(17.3-23.8)
High education	32.4	(28.8-36.1)	55.0	(51.0-58.8)	12.7	(10.3-15.4)
**45-64 Years**	36.4	(34.4-38.4)	49.6	(47.5-51.7)	14.0	(12.7-15.4)
Low education	30.1	(25.5-35.1)	49.9	(44.4-55.5)	20.0	(16.2-24.5)
Medium education	36.2	(33.3-39.1)	49.1	(46.2-52.1)	14.7	(12.9-16.8)
High education	38.9	(36.3-41.5)	50.6	(48.1-53.1)	10.5	(9.0-12.2)
**≥ 65 Years**	61.1	(58.9-63.2)	33.2	(31.1-35.4)	5.7	(4.7-6.8)
Low education	63.5	(57.9-68.7)	30.0	(25.3-35.3)	6.5	(4.3-9.6)
Medium education	59.4	(56.3-62.4)	34.2	(31.1-37.4)	6.4	(5.0-8.2)
High education	63.1	(59.7-66.3)	32.9	(29.7-36.2)	4.0	(3.0-5.5)
**Total (women and men)**	**46.3**	**(45.4-47.3)**	**43.1**	**(42.1-44.0)**	**10.6**	**(10.0-11.3)**

CI=confidence interval
